# Development, behavior, and biomarker characterization of Smith-Lemli-Opitz syndrome: an update

**DOI:** 10.1186/s11689-016-9145-x

**Published:** 2016-04-05

**Authors:** Audrey Thurm, Elaine Tierney, Cristan Farmer, Phebe Albert, Lisa Joseph, Susan Swedo, Simona Bianconi, Irena Bukelis, Courtney Wheeler, Geeta Sarphare, Diane Lanham, Christopher A. Wassif, Forbes D. Porter

**Affiliations:** Pediatrics and Developmental Neuroscience Branch, National Institute of Mental Health, Bethesda, MD 20892 USA; Kennedy Krieger Institute, 716 N. Broadway, Baltimore, MD 21205 USA; Division of Intramural Research, Eunice Kennedy Shriver National Institute of Child Health and Human Development, Bethesda, MD 20892 USA; Department of Psychiatry and Behavioral Sciences, Johns Hopkins University School of Medicine, Baltimore, MD 21205 USA

**Keywords:** Smith-Lemli-Opitz, Autism, Developmental delay, Sterols

## Abstract

**Background:**

Smith-Lemli-Opitz syndrome (SLOS) is an autosomal recessive inborn error of cholesterol metabolism syndrome with neurocognitive manifestations. SLOS is the result of mutations in the gene encoding the 7-dehydrocholesterol reductase, which results in the elevation of the cholesterol precursor 7-dehydrocholesterol (7-DHC). Previous reports indicate that intellectual disability, behavioral disturbances, and autism symptoms are frequently part of the SLOS behavioral phenotype. In the current study, we characterize the developmental history and current behavior of 33 individuals with SLOS aged 4 to 23 years and report on biomarkers 7-DHC and 8-DHC in relation to cognition and behavior.

**Methods:**

This was an observational case series, wherein participants with SLOS underwent extensive behavioral evaluation of cognitive function, adaptive function, autism symptoms, and problem behaviors, in addition to parent report of developmental milestones. Serum and CSF were contemporaneously obtained from the majority of participants.

**Results:**

Developmental milestones such as walking, talking, and toileting were uniformly delayed. Overall levels of cognitive and adaptive functioning were low; no participant received adaptive behavior scores in the average range, and the mean level of cognitive functioning in the full sample was in the moderate range of impairment. Aggressive behavior was present in nearly half of participants. Although the majority of participants had elevated scores on the gold standard autism diagnostic instruments, only about half of participants received a clinical diagnosis of autism spectrum disorder. Finally, while CSF cholesterol was not found to correlate with cognitive or adaptive functioning, both serum and CSF 7-DHC and 8-DHC (and their ratios with cholesterol) were moderately and negatively correlated with functioning in this group.

**Conclusions:**

A history of developmental delay, followed by intellectual disability, is common in individuals with SLOS. Although autism spectrum disorder appears to be a frequent diagnosis in this population, it is apparent that the low level of functioning observed in SLOS may artificially inflate scores on standard autism assessments. Our findings further support that cholesterol precursors 7-DHC and 8-DHC are important biomarkers of the level of functioning in SLOS, especially regarding cognitive abilities, and thus may be to explore as mediators within the context of treatment trials.

**Trial registration:**

ClinicalTrials.gov, NCT00001721, NCT00064792

**Electronic supplementary material:**

The online version of this article (doi:10.1186/s11689-016-9145-x) contains supplementary material, which is available to authorized users.

## Background

Smith-Lemli-Opitz syndrome (SLOS; OMIM #270400) is a biochemically defined syndrome with neurocognitive and developmental manifestations. In SLOS, endogenous cholesterol synthesis has been impaired at the penultimate step of the conversion of 7-dehydrocholesterol (7-DHC) to cholesterol, resulting in lowered serum cholesterol levels and elevated cholesterol precursor 7-DHC [1, 2]. SLOS is autosomal recessive and is caused by a mutation of the *DHCR7* gene (located on chromosome 11q12-13) [3]. The estimated incidence of SLOS is 1 in 39,000 births [4].

SLOS is diagnosable during the fetal period, with diverse presentations of specific dysmorphic features reported in the literature [1, 5]. Among them are cutaneous syndactyly of the second and third toes, post-axial polydactyly of the hands or feet, cleft palate, abnormal gingivae, and hypospadias. Other dysmorphic features may include microcephaly, ptosis, epicanthal folds, short nasal bridge, bitemporal narrowing, and micrognathia. Less commonly reported features include hammertoes, dorsiflexed halluces, cutaneous syndactyly on the fingers, and clinodactyly, as well as medical problems affecting multiple systems [5].

### Cognitive and language development in SLOS

Developmental delay and behavioral problems were reported early on in the description of SLOS [6]. Tierney et al. [7] conducted the largest behavioral phenotyping study of the condition, which included 56 subjects that ranged in age from infancy to age 32. Only about half of those subjects had mental ages above 18 months. With respect to language, as measured by the MacArthur Child Development Inventory [8], the majority of participants (78 %) were found to have expressive language skills equivalent to a child under 30 months, with 43 % under 8 months. Another group conducted cognitive and behavioral testing on a sample of 14 subjects with SLOS aged 3–16, finding cognitive scores that ranged from 28 to 75 [9].

### Behavior problems and autism spectrum disorder

Previous reports indicated that individuals with SLOS engage in multiple types of maladaptive behaviors, including frequent self-injury and other stereotypic movements. In the Tierney et al. phenotyping study, half of the 17 participants with Autism Diagnostic Interview-Revised (ADI-R; [10]) administrations exceeded the cutoff for autism derived from this measure. Of note, the percentage above the cutoff was lower (22 %) for those who had already started cholesterol supplementation by age 5 (age at which diagnostic criteria are assessed per history reported on the ADI-R), compared to those who started after age 5 (88 %) [7].

In a subsequent study by Sikora et al., 71 % of 14 individuals with SLOS met the criteria for a diagnosis of autistic disorder or pervasive developmental disorder, not otherwise specified based on completion of a semi-structured interview informing the Diagnostic and Statistical Manual for Mental Disorder, 4th edition (DSM-IV) checklist [9]. This study also employed the Autism Diagnostic Observation Schedule (ADOS) [11] and the Gilliam Autism Rating Scale [12]; it is worth noting that the rate of classification of autism spectrum disorder (ASD) varied by scale. Eighty-six percent were above the cutoff on the ADOS, while only 21 % exceeded the cutoff on the Gilliam.

The specificity of the gold standard measures of ASD symptoms (ADI-R and ADOS) is low in individuals with severe and profound intellectual disability (ID) [13, 14], and these measures must therefore be used with caution in individuals with a nonverbal mental age (NVMA) of less than 18 months and/or co-occurring physical or sensory disabilities. This appears especially true in SLOS, as a substantial proportion of individuals have severe or profound ID and associated neurological features that affect physical and sensory functioning.

### Biomarkers of SLOS and behavior

Studies have begun exploring phenotypes in relation to biochemical manifestations of the disorder. Specifically, the relationships among markers of abnormal sterol metabolism and aspects of the behavioral phenotype in SLOS have now been examined in several reports [9, 15, 16]. Sikora et al. examined correlations of autism symptom measures with cholesterol, 7-DHC, and 8-DHC and found no significant correlations [9]. Another recent study tested whether the ratio of 7-DHC + 8-DHC to cholesterol was related to IQ and/or challenging behavior in a small sample (*n* = 13) of children diagnosed with SLOS. This study did not find a relationship of sterol levels with IQ but did find a relationship between some specific maladaptive behaviors (described as biobehavioral) and the ratio of combined 7-DHC and 8-DHC to cholesterol [15].

Sparks et al. [16] measured the levels of serotonin and dopamine metabolites, 5-hydroxyindoleacetic acid (5HIAA), and homovanillic acid (HVA) in the cerebral spinal fluid (CSF) of 21 individuals with SLOS in an attempt to correlate anatomical severity scores, Aberrant Behavior Checklist scores, and concurrent sterol biochemistry. No evidence was found for correlation among CSF 5HIAA, HVA, 7-DHC, and 8-DHC, nor were correlations found with the behavioral measures.

The goal of the current study was to provide an updated description of cognitive and behavioral characteristics of children and adolescents with SLOS. Emphasizing early development, cognitive and adaptive functioning, and recently updated diagnostic changes and algorithms for diagnostic measures of autism [17], we were interested in exploring associated symptoms while accounting for the global developmental delays and medical problems found in this population. We also sought to determine whether serum or CSF cholesterol, 7-DHC, or 8-DHC serve as useful biological markers of cognitive impairment in SLOS.

## Methods

### Participants

This study describes a subset of 33 research participants with SLOS who underwent the full complement of behavioral assessments, taken from a longitudinal natural history study of SLOS (NCT00001721) or from the baseline visit of a clinical trial of simvastatin in individuals with SLOS (NCT00064792) (Table 1). The diagnosis of SLOS was confirmed biochemically and molecularly for all patients. Two of these patients were previously described in Tierney et al. [7]. The behavioral assessment battery was completed in 1–2 days; when more than one behavioral assessment was available from the natural history study, the earliest visit was used. Children from families primarily using languages other than English were excluded from this analysis because measures were administered in English.

**Table 1:** Summary of patient characteristics

	KKI	NIH	Full sample
*n*	22	11	33
Male, *n* (%)	13 (59 %)	6 (55 %)	19 (58 %)
Age, years (M ± SD)	8.04 ± 3.63	10.84 ± 6.29	8.98 ± 4.78
Anatomical severity (M ± SD)	13.45 ± 7.22	11.73 ± 5.12	12.88 ± 6.56
Simvastatin, *n* (%)	0	1 (9 %)	1 (3 %)
Cholesterol supplementation, *n* (%)	22 (100 %)	8 (73 %)	30 (91 %)
Clinical judgment of autism diagnosis			
ASD	15 (68 %)	3 (27 %)	18 (55 %)
Nonspectrum	7 (32 %)	8 (73 %)	15 (45 %)
ADOS classification			
Autism	16 (73 %)	4 (36 %)	20 (61 %)
Autism spectrum	2 (9 %)	2 (18 %)	4 (12 %)
Nonspectrum	4 (18 %)	5 (45 %)	9 (27 %)
ADI-R classification			
Autism	11 (50 %)	8 (73 %)	19 (58 %)
Nonspectrum	11 (50 %)	3 (27 %)	14 (42 %)
Vineland-II Adaptive Behavior Composite (M ± SD)	49.18 ± 17.56	58.82 ± 12.94	52.39 ± 16.60
Nonverbal IQ (M ± SD)	47.45 ± 24.22	45.15 ± 17.56	46.68 ± 21.96
Verbal IQ (M ± SD)	39.44 ± 30.23	41.50 ± 17.86	40.17 ± 26.19
Full-scale IQ (M ± SD)	44.20 ± 25.26	42.41 ± 16.75	43.57 ± 22.33

Note: IQ was estimated with the Mullen Scales of Early Learning (KKI, *n* = 8; NIH, *n* = 6) or the Stanford-Binet Scales of Intelligence (KKI, *n* = 15; NIH, *n* = 5). Two individuals (KKI) were each missing VIQ and FSIQ. ASD refers to any of the DSM-IV pervasive developmental disorders

### Ethics, consent, and permissions

Families from across North America participated in studies that occurred either at the National Institutes of Health (NIH) (Bethesda, MD) or at the Kennedy Krieger Institute (Baltimore, MD), based on the protocols approved by the institutional review boards of the Johns Hopkins Medical Institutions and the *Eunice Kennedy Shriver* National Institute for Child Health and Human Development. Informed consent and assent, if applicable, was obtained for all subjects.

### Neurocognitive and behavioral assessment

A standard battery was administered to each participant, including assessments of cognitive ability, adaptive behavior, problem behaviors, and symptoms of autism spectrum disorder. Given the range of ability in this cohort, no single cognitive test was appropriate for all participants. The Stanford-Binet Intelligence Test [18] was attempted; if the participant was unable to achieve the basal score on at least four subtests, then the Mullen Scales of Early Learning [19] was administered. Full-scale IQ (FSIQ), nonverbal IQ (NVIQ), and verbal IQ (VIQ) are reported for the Stanford-Binet; for comparability (and to accommodate out-of-age-range testing), the corresponding scores were calculated from the Mullen using developmental quotients (DQ; mental age divided by chronological age). For the Mullen, NVDQ was calculated from the nonverbal mental age, the average of the Visual Reception and Fine Motor subscales, divided by chronological age, while VDQ was calculated from the verbal mental age, the average of the Expressive and Receptive Language subscales, divided by chronological age. Full scale was the average of NVDQ and VDQ. Hereafter, we use the term IQ to refer to both IQ and DQ scores. IQ scores have a mean of 100 and a standard deviation of 15; by convention, IQ scores are described using the following categories: ≥85 average, ≥70 borderline, ≥55 mild impairment, ≥40 moderate impairment, ≥25 severe impairment, and <25 profound impairment.

Adaptive behavior was assessed with the parent report form of the Vineland Adaptive Behavior Scales, 2nd edition [20]. The Vineland-II yields standard scores (mean = 100, standard deviation = 15) on four domains: Communication, Daily Living Skills, Socialization, and Motor Skills (for subjects younger than 8 years), as well as the Adaptive Behavior Composite. Scores less than 70 are considered impaired (70–79 borderline; 80–89 below average; 90+ average).

Finally, the gold standard autism diagnostic battery was administered: the play-based Autism Diagnostic Observation Schedule (ADOS; [11]), the semi-structured parent interview Autism Diagnostic Interview-Revised (ADI-R; [10]), and the clinical judgment of the DSM-IV-TR criteria. Both the ADOS and ADI-R are diagnostic instruments that produce total and symptom domain scores, which are evaluated against a cutoff for suspicion of autism (higher threshold) or autism spectrum (lower threshold, ADOS only). Using algorithms modified in 2007, the ADOS also produces calibrated severity scores (CSS), which standardize scores according to age and language ability and range from 1 to 10 [17]. The ADI-R is an extensive interview that covers past and current behaviors, as well as information about developmental milestones and problem behaviors. Standard procedures were followed for achieving and maintaining research reliability on the ADI-R and the ADOS. Both the ADI-R and the ADOS were administered for research purposes here, regardless of the participants’ mental age. It should be noted that the ADI-R suggested nonverbal mental age requirement is 24 months and caution should be used for interpreting both the ADI-R and ADOS in individuals with low nonverbal mental age.

Clinical judgment was completed by doctoral-level clinicians with extensive experience in assessing individuals with intellectual disability and the diagnosis of autism spectrum disorder, using all available information and basing their judgment on a checklist of the Diagnostic and Statistical Manual for Mental Disorder, 4th edition (DSM-IV; [21]) criteria for autistic disorder and pervasive developmental disorder, not otherwise specified (PDD-NOS). Hereafter, we refer to either autistic disorder or PDD-NOS with the omnibus term, autism spectrum disorder (ASD), consistent with the autism spectrum disorder term included in the 2013 publication of the Diagnostic and Statistical Manual for Mental Disorder, 5th edition (DSM-5; [22]) (although participants in the current study were not evaluated with the DSM-5 criteria).

### Other evaluations

Concurrent blood samples were collected from the majority (*n* = 30) of participants. CSF was obtained contemporaneously on a subset (*n* = 26) of those participants. With one exception of a 12-day lag, all samples and data were collected during a 5-day admission to the NIH Clinical Center and/or the Kennedy Krieger Institute, during which time the behavioral assessments were also conducted. Both serum and CSF were analyzed for levels of cholesterol, 7-DHC, and 8-DHC. Severity of anatomical abnormality was rated using the severity scale developed by Bialer and later refined by Kelley et al. [23], based on categories of brain, oral, acral, eye, heart, kidney, liver, lung, bowel, and genitalia abnormalities. Possible scores range from 0 to 100, as ratings that range from 0 to 2 for each item are normalized.

## Results

Participant characteristics are summarized in Table 1. Individual results of the behavioral evaluation are shown in Table 2. The 33 participants ranged in age from 4 to 23 years (M ± SD = 8.98 ± 4.78), and severity scores ranged from 6 to 33. All but three participants were reported to currently take cholesterol supplementation.

**Table 2:** Cognitive, adaptive behavior, and ASD status

ID	Sex	Hearing imp.	Age at visit	Anatomical severity	Age of walking	VABS-II ABC	NVIQ	VIQ	FSIQ	IQ test	ADOS class.	ADI-R class.	Clinical judgment
K01	M	--	7.13	11	n/a	32	42	43	40	SB	AUT	AUT	ASD
K04	M	--	8.67	22	48	29	10.1	12.0	11.1	MSEL	AUT	AUT	ASD
K05	F	N	17.53	17	60	19	9.5	8.1	8.8	MSEL	AUT	AUT	ASD
N09	M	N	23.27	11	24	61	66	68	66	SB	AUT	AUT	ASD
K12	M	N	9.08	6	18	71	56	77	65	SB	NS	NS	NS
K13	M	N	10.61	17	24	53	60	3.2	56	SB	AUT	NS	ASD
K18	F	N	12.02	22	48	34	42	43	40	SB	AUT	NS	ASD
K24	F	Y	7.58	11	24	50	46	--	--	SB	AUT	AUT	ASD
K25	F	--	10.08	11	12	60	60	49	52	SB	NS	NS	NS
K26	M	--	13.32	6	42	45	51	49	48	SB	AUT	AUT	ASD
K33	M	Y	5.58	33	n/a	43	33.6	17.9	25.8	MSEL	AUT	AUT	ASD
K34	M	--	5.68	11	22	32	17.6	8.8	13.2	MSEL	AUT	NS	ASD
N36	F	N	11.06	6	18	50	18.6	20.5	19.5	MSEL	AUT	AUT	NS
N45	M	Y	10.63	6	17	75	62	59	59	SB	NS	NS	NS
K55	F	--	4.41	11	15	69	53	56	52	SB	AUT	AUT	ASD
K56	F	N	6.09	6	30	78	74	93	83	SB	NS	NS	NS
N58	F	Y	8.05	22	n/a	72	60	56	56	SB	NS	NS	NS
K60	M	N	4.74	11	20	67	70	--	--	SB	AS	AUT	ASD
K61	M	Y	6.30	17	20	63	66	59	61	SB	NS	NS	NS
K62	F	Y	10.30	11	28	54	59	1.8	51	SB	AUT	NS	NS
K63	F	--	5.41	22	54	36	20.8	18.5	19.7	MSEL	AUT	AUT	ASD
K64	M	N	4.03	6	17	66	69	83	75	SB	AUT	NS	NS
K66	M	Y	4.08	11	18	72	103	86	94	SB	AUT	NS	NS
K68	F	Y	4.08	11	55	53	64	57	58	SB	AS	AUT	ASD
K70	M	--	13.25	11	21	25	15.1	12.6	13.9	MSEL	AUT	NS	ASD
N71	M	--	4.97	17	36	62	53.4	55.1	54.2	MSEL	NS	AUT	NS
N72	M	--	9.73	11	18	60	43	43	40	SB	AS	AUT	ASD
K73	M	N	6.96	6	30	31	22.3	10.8	16.6	MSEL	AUT	AUT	ASD
N78	F	--	20.99	17	36	36	42	43	40	SB	AS	AUT	NS
N79	F	--	13.63	22	96	37	11.7	8.3	10.0	MSEL	AUT	AUT	NS
N86	F	N	3.95	6	24	70	58.5	29.8	44.1	MSEL	NS	NS	NS
N87	M	N	7.49	6	24	61	37.6	31.5	34.6	MSEL	NS	AUT	ASD
N97	M	--	5.49	11	23	63	43.8	42.3	43.1	MSEL	AUT	AUT	NS

Note. ID indicates site; those beginning with K were seen at the KKI, and those beginning with N were seen at the NIH. IQ with one decimal place indicates developmental quotient (mental age divided by chronological age) used, while scores with no decimal places indicate IQ used. Double dash (--) indicates missing data. Only one subject (N09) was on simvastatin at the time of evaluation. All subjects except N78, N79, and N87 were taking cholesterol supplementation at evaluation. n/a for age of walking indicates that the child was not yet walking at evaluation; ages are in months

*Hearing imp* hearing impairment, *VABS-II ABC* Vineland-II Adaptive Behavior Composite, *NVIQ* nonverbal IQ, *VIQ* verbal IQ, *FSIQ* full-scale IQ, *ADOS* Autism Diagnostic Observation Schedule, *ADI-R* Autism Diagnostic Interview, Revised, *SB* Stanford-Binet Intelligence Scales, *MSEL* Mullen Scales of Early Learning, *NS* nonspectrum, *AS* autism spectrum, *AUT* autism, *ASD* autism spectrum disorder

### Attainment of developmental milestones

Information on developmental milestones was obtained from the ADI-R. Generally, milestones were delayed. Notable, however, was the wide age range during which attainment of milestones occurred; children were generally taking first steps and using first words in mid-to-late childhood. Walking was delayed in most participants; only four (12 %) individuals walked prior to 18 months, an additional 19 (58 %) participants walked by 36 months, seven (21 %) participants walked between 36 and 96 months, and three (9 %) subjects (aged 5–7 years) were not yet walking at the time of evaluation. Toilet training data was missing for five participants; among the remaining 28 with data, nighttime bladder control was achieved between 42 and 138 months (*n* = 11, 39 %) or not at all (*n* = 17, 61 %). Ten (36 %) participants had achieved daytime bladder control, at ages ranging from 42 to 144 months. Those same ten subjects had also achieved bowel control (age of attainment range from 42 to 138 months).

Language milestones were also delayed; only ten participants (30 %) used their first words by 24 months. The remainder were either not speaking at the time of evaluation (*n* = 4, 12 %) or first used language between 27 and 121 months (*n* = 19, 58 %). One third (*n* = 11) of participants were not using phrase speech at the time of evaluation; among the remaining, only five (15 %) used phrase speech prior to 33 months. The age of first use of phrases ranged up to 126 months. A loss of language skills was reported for only three (9 %) participants; these losses were reported to occur in late toddlerhood (33 and 35 months) or mid-to-late childhood (63 months). Those who lost language in toddlerhood were reported to have not yet regained those skills (at age 7 and 20 years, respectively), while the loss in later childhood lasted for less than 1 year. No loss was reported to coincide with an illness.

### Cognitive and adaptive functioning

Participants received either the Stanford-Binet (*n* = 20, 61 %) or the Mullen Scales of Early Learning (*n* = 13, 39 %) as a test of cognitive functioning. Eight (62 %) of the 13 participants who received the Mullen were out of age range for the test (which is standardized up to 5 years, 8 months). Two participants received only the nonverbal scales, so for the purposes of summarizing cognitive function in this paragraph, their full-scale scores were imputed at those values. The majority (*n* = 30, 91 %) of participants had a nonverbal mental age greater than 18 months, and *n* = 26 (78 %) had a nonverbal mental age greater than 24 months. The mean level of functioning in the full sample was in the moderate range of impairment (mean FSIQ, 44.44 ± 22.11), but the full range of impairment categories was represented: profound (*n* = 8, 24 %), severe (*n* = 2, 6 %), moderate (*n* = 12, 36 %), mild (*n* = 7, 21 %), borderline (*n* = 3, 9 %), and average (*n* = 1, 3 %). Among the 31 participants with both NVIQ and VIQ, the average split (NVIQ-VIQ) was 5.78 ± 16.70 and ranged from −21 to +57. Typically, a split of 10 points is considered clinically significant, and this was observed in ten (30 %) participants (*n* = 3, VIQ > NVIQ; *n* = 7, NVIQ > VIQ).

Vineland-II scores were consistent with the cognitive testing, in that mean scores in all domains were in the impaired range of functioning. No participant had an Adaptive Behavior Composite score in the average range (M ± SD = 52.39 ± 16.60, range 19–78). Mean subscale scores were lowest for Daily Living Skills (M ± SD = 50.21 ± 19.75), followed by Communication (M ± SD = 55.64 ± 19.90) and Socialization (M ± SD = 62.94 ± 16.69). The mean Motor Skills score, assessed only in participants 7 years of age or younger (*n* = 24), was also in the impaired range (M ± SD = 60.21 ± 23.44).

### Behavioral characterization

#### ASD symptom measurement

Most participants received an ADOS Module 1 (*n* = 18, 55 %), based on single-word-level speech; eight (24 %) received a Module 2, based on observed phrase speech; six (18 %) received a Module 3, and one (3 %) received a Module 4, both used when the individual is capable of fluent speech. Most ADOS total scores exceeded the threshold for autism (*n* = 20, 61 %); four (12 %) participants had scores in the autism spectrum range, and nine (27 %) were in the nonspectrum range (below both cutoffs). CSS were available for *n* = 31 participants; the mean CSS was 5.39 ± 2.20, which is considered to be in the autism spectrum range.

Scores on the ADI-R exceeded the autism cutoffs for 19 (58 %) participants. On average, participants who exceeded the autism threshold on either the ADI-R or the ADOS had greater cognitive impairment than those who did not. Although the CSS did not significantly correlate with age or NVIQ, the average NVIQ in the participants with above autism spectrum-threshold ADOS scores was 42.21 ± 23.68, compared to 58.61 ± 9.89 (*F* = 3.99, *p* = .055) in participants with below-threshold scores. Stratification by ADI-R classification yielded similar results: 38.86 ± 18.97 in participants who exceeded the autism threshold versus 57.30 ± 21.88 (*F* = 6.69, *p* = .01) in those below.

Based on clinical judgment of all 33 participants, 55 % (*n* = 18) were assigned an ASD diagnosis. A nonsignificant site difference did emerge in clinical judgment; participants evaluated at the NIH were less likely to be diagnosed with ASD (NIH = 27 %, KKI = 68 %; Fisher’s exact test, *p* = .06). Rates of exceeding the threshold on the ADOS (NIH = 55 %, KKI = 82 %; Fisher’s exact test, *p* = .12) and ADI-R (NIH = 73 %, KKI = 50 %; Fisher’s exact test, *p* = .28) were more similar between sites. Six of the seven participants with nonverbal mental age less than 24 months had a final clinical judgment of ASD (and were all seen at Kennedy Krieger Institute (KKI)). The mean NVIQ in the participants with clinical judgment of ASD (39.09 ± 19.64) was significantly lower than in those with clinical judgment of nonspectrum (55.80 ± 21.71) (*F* = 5.39, *p* = .03).

#### Problem behaviors

Item-level ADI-R data were available for 28 (85 %) participants; the percentages in this section use these 28 individuals as the denominator. There was no systematic reason for the missing data; three participants were in the profound range of functioning, and three were in the borderline-to-average range. According to parent report, half (*n* = 14) of participants currently engaged in definite aggression towards family members/caregivers, and eight of those participants were also aggressive towards nonfamily members. Seven (25 %) participants were reported to currently engage in significant self-injury resulting in tissue damage; five of those participants were also aggressive towards family or nonfamily members. Current aggression was not significantly related to the level of cognitive impairment; the mean FSIQ was slightly lower in participants reported to engage in either type of aggression (39.46 ± 19.07 versus 45.47 ± 16.69; *F* = 0.79, *p* = .38), and a slightly larger proportion of those who demonstrated aggressive behavior were classified as severe/profound (36 % versus 21 %; Fisher’s exact test, *p* = .68). A similar pattern was observed for self-injurious behavior.

### Serum and CSF cholesterol and sterol levels

Descriptive statistics for serum and CSF cholesterol and DHC levels are presented in Table 3 (individual serum and CSF cholesterol and DHC levels are found in Additional file 1: Table S1). The correspondences between CSF and serum cholesterol levels were nonsignificant for cholesterol (*r* = .22, *p* = .28) and 8-DHC (*r* = .16, *p* = .42) but moderate and significant for 7-DHC (*r* = .50, *p* = .01). Age did not correlate with serum or CSF cholesterol or serum 7-DHC or 8-DHC, though there were moderate and significant correlations between age and CSF 7-DHC and 8-DNC (Table 3). Thus, we elected to proceed with caution and calculated partial correlations between phenotypic measures and cholesterol/sterol levels, controlling for age (Table 3). CSF cholesterol was not significantly correlated with Vineland-II or IQ scores. However, serum cholesterol was moderately and positively correlated with Vineland-II and IQ scores, while both serum and CSF DHC levels were moderately and negatively correlated with Vineland-II and IQ. The 7-DHC + 8-DHC to cholesterol ratio in both serum and CSF was significantly and moderately-to-strongly related to all Vineland-II and IQ variables, and serum was significantly correlated with anatomical severity score. None of the markers were significantly correlated with the ADOS calibrated severity score. In summary, both CSF and serum dehydrosterols, especially the ratio of 7-DHC + 8-DHC to cholesterol, performed as biomarkers for general cognitive and adaptive function.

**Table 3:** Serum and CSF cholesterol and sterol levels

	M ± SD	Range	Age	Comm	DLS	Soc	Motor	ABC	NVIQ	VIQ	FSIQ	Anatomical severity
			*r*	*r*_p_								
Serum, mg/dL (*n* = 30)												
Cholesterol	108.90 ± 32.39	(47–181)	−.19	.45*	.51**	.44*	.26	.53**	.45*	.23	.35	−.19
7-DHC	5.70 ± 4.93	(0.044–19.00)	.23	−.25	−.24	−.47**	−.51*	−.31	−.50**	−.30	−.48*	.23
8-DHC	5.48 ± 5.29	(0.14–28.00)	.11	−.14	−.07	−.29	−.29	−.16	−.36	−.23	−.35	.11
Ratio	0.11 ± 0.10	(0.0014–0.39)	.25	−.43*	−.44*	−.59**	−.62**	−.50**	−.65**	−.41*	−.62**	.38*
CSF, μg/mL (*n* = 26)												
Cholesterol	1.83 ± 0.58	(0.96–3.66)	.02	−.07	−.13	.02	.03	−.06	.14	−.08	.07	−.19
7-DHC	0.034 ± 0.028	(0–0.097)	.47*	−.56**	−.54**	−.52**	−.68**	−.58**	−.52**	−.32	−.51*	.24
8-DHC	0.072 ± 0.043	(0.006–0.17)	.39*	−.48*	−.43*	−.32	−.47*	−.46*	−.40*	−.30	−.38	.18
Ratio	0.065 ± 0.046	(0.0028–0.15)	.38	−.53**	−.52**	−.51**	−.66**	−.57**	−.59**	−.34	−.56**	.32

Note: *r* = Pearson correlation; *r*_p_ = partial Pearson correlation, controlling for age. Ratio is the ratio of combined 7-DHC and 8-DHC, divided by cholesterol concentration. Sample sizes vary slightly due to missing data; missing *n* = 2 VIQ and *n* = 2 FSIQ. Vineland-II motor scores are available only through 7 years of age; thus, the sample size for serum correlations was *n* = 23 and for CSF correlations *n* = 22

*p < .05; **p < .01

## Discussion

Findings from this study are consistent with previous reports indicating cognitive and adaptive behavior is significantly impaired in SLOS. Similar to other studies, almost one third of SLOS participants were in the severe to profound range of ID, and only one participant had average scores. Further, exploration of early developmental skill attainment indicated motoric, daily living skills, and language milestones were almost all universally delayed. However, the age range of attainment was wide and extraordinarily late in several cases (e.g., walking attainment up to age 8, language onset up to age 10). Thus, the general pattern of development is best characterized as one of delay (or stagnation) rather than deviance, given that most participants achieved milestones, albeit in mid-to-late childhood. This finding has important implications for intervention studies, because children may be expected to spontaneously develop new skills, even far outside of what is considered the typical window.

With respect to autism symptomatology, several findings of this study are of interest. While the rate of ASD in this study was similar to that in other published work (53–71 %) [7, 9], this rate should be interpreted with caution. First, all estimates of ASD rate in SLOS are necessarily based on small samples, so the 95 % confidence intervals surrounding the published point estimates are extremely wide. Second, while a diagnosis of autism was common in this SLOS sample, receiving an ASD diagnosis was significantly related to NVIQ (ASD < non-ASD). This was borne out in the between-site differences in the rate of ASD diagnosis, in that KKI patients were generally lower functioning. Six of the seven individuals with a mental age below 24 months were evaluated at the KKI, and all of these children were given a clinical diagnosis (using the DSM-IV criteria for autistic disorder or PDD-NOS) of ASD. Caution is thus warranted with respect to whether the DSM-5 criteria would be met.

Specifically, while this study used the DSM-IV criteria for autism, only a few of the DSM-IV criteria for autistic disorder explicitly included consideration of developmental level. The DSM-5 specifically includes criterion E, which states “These disturbances are not better explained by intellectual disability or global developmental delay.” While this criterion is new, the need for such consideration has been noted for some time; Moss and Howlin [24] urged extra caution when parsing out clinically relevant symptoms of autism from intellectual disability in individuals with genetic conditions. While this clearly pertains to individuals with mental ages below 24 months, significant motor impairments and other congenital problems appear to complicate the picture to extend these concerns to higher mental ages as well.

The weaknesses of the diagnostic measures for teasing out these factors is reflected in the sensitivity and specificity rates for how cutoffs from these measures compare to clinical judgment of ASD. Similar to other studies that have included individuals with profound intellectual disability [13], specificity of these measures was generally low (53 % for ADOS, and 67 % for ADI-R), although we did note higher specificity of the measures in the KKI sample (ADI-R 100 %, ADOS 57 %) compared to the NIH sample (ADI-R 37.5 %, ADOS 50 %), which may have indicated differences in the interpretation of these measures.

These results were recently supported by a large meta-analysis of several other genetic disorders associated with ASD (but not SLOS), in the finding that autism spectrum classification is often related to the degree of intellectual disability in genetic disorders associated with ID [25]. Although the sample size and heterogeneity in the current study prohibit an idiopathic autism comparison, the findings that individuals who had higher ADOS and ADI-R scores had lower IQ is supported by observations from other specific genetic disorders associated with ASD, such as fragile X [26, 27]. Within these genetic disorders, clinically significant ASD symptomatology appears to dissociate less from cognitive impairment than it does in idiopathic ASD [28]. In SLOS and other disorders with other systemic medical problems, this lack of dissociation with IQ may also relate to the disproportionately delayed early milestone attainment and related sensory impairments and medical problems, which may mask as deviancy instead of delay, compared to pure idiopathic autism [29, 30].

With respect to other behavioral problems frequently reported in SLOS, this study found high rates of aggression and self-injury, although not as high as was recently reported using a different measure [15]. In that study, all 13 children with SLOS were reported to engage in aggression towards others, self-injurious behaviors, and destruction of property. The average age of that sample was about 5 years, considerably younger than the current study. In the largest phenotyping study, Tierney et al. reported a history of aggression in 63 % and history of self-injurious behaviors in 89 % of participants with SLOS [7]. Similarly high rates of aggression (52 %) and self-injurious behavior (35 %) were reported in a cohort from the UK [31]. While there is a great deal of variation in the rate of aggression in many genetic disorders associated with intellectual disability [32], within SLOS, the methodological differences may be a factor in the differences found between the current and previous studies. The ADI-R assessment of aggressive and self-injurious behaviors was characterized more broadly and included a history of the behavior in Tierney et al. [7] while the measure used by Freeman et al. [15] was broadened by including property destruction. Differences from earlier studies may also be attributable to the history of cholesterol supplementation; supplementation at a young age is more common now than it would have been for the individuals reported on in previous studies.

Serum sterols (cholesterol and dehydrosterol measurements) and CSF dehydrosterols and their ratio to cholesterol appear to be linearly related to impairment in cognitive and adaptive functioning. As such, cognitive and adaptive functioning may be useful as outcome measures in any therapeutic trial. One other study demonstrated a relationship between the dehydrosterol to cholesterol ratio and problem behavior (though not IQ) [15]; in the current study, we found moderate-to-strong relationships between the sterols (and especially the ratios) and measures of cognitive and adaptive function. Correlations of both 7-DHC and 8-DHC were generally stronger in the CSF compared to serum, consistent with the blood-brain barrier separation of peripheral and brain cholesterol metabolism. Correlations with functioning were strongest with 7-DHC, consistent with previous findings that congenital abnormalities and total severity score increase as residual enzyme activity decreased and the levels of 7-DHC increased [33–35]. It is also of note that correlations for both cholesterol precursors were highest with cognitive and adaptive areas requiring significant motor skills.

The fact that these biomarkers correlated with contemporaneous measures of cognitive and adaptive functioning has implications for using the biomarker approach to the measurement of change in treatment trials. Specifically, this study provides evidence that accumulation of the aberrant sterol 7-DHC in the brain is related to cognitive and adaptive functioning. Thus, these sterol levels may truly be biomarkers that can aid in predicting changes in cognition and behavior in response to treatment. In addition, based on a previous study, the relationship of these biomarkers to outcomes may also be related to their relationship to severity of structural brain abnormalities in this population [36].While cognitive and behavioral improvements are the end goals for treatment trials of conditions associated with ID and ASD, very few conditions associated with these disorders currently have the potential to use such a biomarker to test mediation of response to treatment [37].

### Study limitations

In order to maximally capture the natural history and current function of SLOS, an untreated sample would be ideal, but is not feasible. Interpretation of our findings requires acknowledgement that the majority of this cohort received cholesterol supplementation during their study participation. However, as cholesterol fails to cross the blood-brain barrier to an appreciable degree [38, 39], it is unlikely that any significant differences would be noted in a control untreated group. Two other limitations likely had a greater impact on this study. First is the lack of an appropriate severity scale that takes into account the behavioral and cognitive function of these patients. The current scale is based on anatomical characteristics that reflect the congenital abnormalities of these patients, but the ratings are sometimes at odds with the clinical evaluations of some of these patients. The second limitation is the restricted range of SLOS severity of the study cohort; participants from the KKI were drawn from a trial with inclusion criteria that prevented any severely affected patients from enrolling. Patients were between the ages of 4 years and less than 18 years, weighed 10 kg or more, had residual fibroblast enzymatic activity that was 10 % or more of the control value (cholesterol synthesis as a fraction of total sterol synthesis), and had a dehydrocholesterol to cholesterol ratio of 1.0 or less.

## Conclusions

The current study adds to previous literature by demonstrating the utility of 7-DHC and 8-DHC, and their ratio with cholesterol, as biomarkers specific to cognitive and adaptive function in SLOS. Further, we confirm previous findings that the vast majority of individuals with SLOS have ID, although there is a broad range of functioning. Future directions for research in this area include using longitudinal data to test whether sterol levels correlate with functioning over time within subjects, therefore determining whether these biomarkers may be useful indicators of treatment response.

## Additional file

Additional file 1: Table S1. This file contains the raw serum and CSF cholesterol and DHC values for each participant. (DOC 73.5 kb)

## Abbreviations

5HIAA: 5-hydroxyindoleacetic acid
ADI-R: Autism Diagnostic Interview-Revised
ADOS: Autism Diagnostic Observation Schedule
ASD: autism spectrum disorder
CSF: cerebrospinal fluid
CSS: calibrated severity score
DHC: dehydrocholesterol
DSM: Diagnostic and Statistical Manual
FSIQ/DQ: full-scale intellectual quotient/developmental quotient
HVA: homovanillic acid
ID: intellectual disability
KKI: Kennedy Krieger Institute
NIH: National Institutes of Health
NVIQ/DQ: nonverbal intellectual quotient/developmental quotient
SLOS: Smith-Lemli-Opitz syndrome
VIQ/DQ: verbal intellectual quotient/developmental quotient
