# Supplementary material for: Development, behavior, and biomarker characterization of Smith-Lemli-Opitz syndrome: an update
Source: J Neurodev Disord. 2016 Apr 5;8:12. doi: 10.1186/s11689-016-9145-x (PMC4822234; doi:10.1186/s11689-016-9145-x)
Supplement: Additional file 1: Table S1. — This file contains the raw serum and CSF cholesterol and DHC values for each participant. (DOC 73.5 kb) [file 11689_2016_9145_MOESM1_ESM.doc]

Additional file 1: Table S1. Serum and CSF cholesterol and DHC values

|  |  |  | **Serum, mg/dL** | | | | **CSF, μg/mL** | | | |
| --- | --- | --- | --- | --- | --- | --- | --- | --- | --- | --- |
| **ID** | **Sex** | **Age at Visit** | **Cholesterol** | **7-DHC** | **8-DHC** | **Ratio** | **Cholesterol** | **7-DHC** | **8-DHC** | **Ratio** |
| K01 | M | 7.13 | 86 | 9.3 | 6.6 | 0.185 | 1.69 | 0.088 | 0.155 | 0.144 |
| K04 | M | 8.67 | 65 | 9.5 | 8.5 | 0.277 | 1.64 | 0.065 | 0.140 | 0.125 |
| K05 | F | 17.53 | 85 | 13.9 | 9.8 | 0.279 | 1.22 | 0.086 | 0.101 | 0.153 |
| K12 | M | 9.08 | 106 | 2.8 | 3.3 | 0.058 | 1.66 | 0.024 | 0.077 | 0.061 |
| K13 | M | 10.61 | 110 | 2.0 | 2.9 | 0.045 | 2.68 | 0.043 | 0.132 | 0.065 |
| K18 | F | 12.02 | 82 | 4.0 | 4.2 | 0.100 | 1.94 | 0.033 | 0.073 | 0.055 |
| K24 | F | 7.58 | 109 | 5.9 | 5.4 | 0.104 | 1.98 | 0.029 | 0.080 | 0.055 |
| K25 | F | 10.08 | 117 | 4.0 | 2.0 | 0.051 |  |  |  |  |
| K26 | M | 13.32 | 78 | 3.3 | 4.4 | 0.099 | 2.02 | 0.028 | 0.084 | 0.055 |
| K33 | M | 5.58 | 101 | 6.3 | 6.2 | 0.124 | 1.73 | 0.041 | 0.079 | 0.069 |
| K55 | F | 4.41 | 129 | 2.2 | 2.8 | 0.039 | 1.94 | 0.035 | 0.083 | 0.061 |
| K56 | F | 6.09 | 96 | 3.4 | 2.9 | 0.066 | 1.58 | 0.026 | 0.063 | 0.056 |
| K60 | M | 4.74 | 149 | 2.9 | 4.9 | 0.052 | 1.90 | 0.036 | 0.102 | 0.073 |
| K61 | M | 6.30 | 108 | 0.2 | 0.8 | 0.009 | 2.99 | 0.003 | 0.030 | 0.011 |
| K62 | F | 10.30 | 94 | 1.7 | 2.8 | 0.048 | 1.66 | 0.020 | 0.047 | 0.040 |
| K63 | F | 5.41 | 74 | 16.8 | 12.1 | 0.391 | 0.96 | 0.057 | 0.090 | 0.153 |
| K64 | M | 4.03 | 47 | 0.4 | 0.4 | 0.017 | 1.22 | 0.010 | 0.038 | 0.039 |
| K66 | M | 4.08 | 162 | 0.1 | 0.3 | 0.002 | 2.18 | 0.000 | 0.006 | 0.003 |
| K68 | F | 4.08 | 105 | 4.5 | 3.5 | 0.076 | 1.85 | 0.049 | 0.074 | 0.066 |
| K70 | M | 13.25 | 78 | 7.1 | 6.8 | 0.178 | 1.81 | 0.097 | 0.165 | 0.145 |
| K73 | M | 6.96 | 131 | 0.0 | 0.1 | 0.001 | 3.66 | 0.002 | 0.009 | 0.003 |
| N09 | M | 23.27 | 125 | 11.7 | 9.7 | 0.171 |  |  |  |  |
| N36 | F | 11.06 | 72 | 6.5 | 5.0 | 0.160 |  |  |  |  |
| N45 | M | 10.63 | 181 | 2.7 | 3.4 | 0.034 | 1.64 | 0.015 | 0.032 | 0.029 |
| N58 | F | 8.05 | 148 | 0.1 | 0.6 | 0.005 | 2.19 | 0.004 | 0.019 | 0.010 |
| N71 | M | 4.97 | 131 | 4.2 | 3.0 | 0.055 | 1.49 | 0.014 | 0.041 | 0.036 |
| N72 | M | 9.73 | 82 | 7.9 | 7.5 | 0.188 | 1.58 | 0.065 | 0.091 | 0.098 |
| N86 | F | 3.95 | 132 | 8.7 | 7.8 | 0.125 | 1.04 | 0.015 | 0.043 | 0.056 |
| N87 | M | 7.49 | 114 | 9.9 | 8.8 | 0.164 |  |  |  |  |
| N97 | M | 5.49 | 170 | 19.0 | 28.0 | 0.276 | 1.35 | 0.009 | 0.028 | 0.027 |

Note: Ratio is the ratio of combined 7DHC and 8DHC, divided by cholesterol concentration.
